# Supplementary figures and images for: Evolution of tunnels in α/β-hydrolase fold proteins—What can we learn from studying epoxide hydrolases?
Source: PLoS Comput Biol. 2022 May 17;18(5):e1010119. doi: 10.1371/journal.pcbi.1010119 (PMC9140254; doi:10.1371/journal.pcbi.1010119)

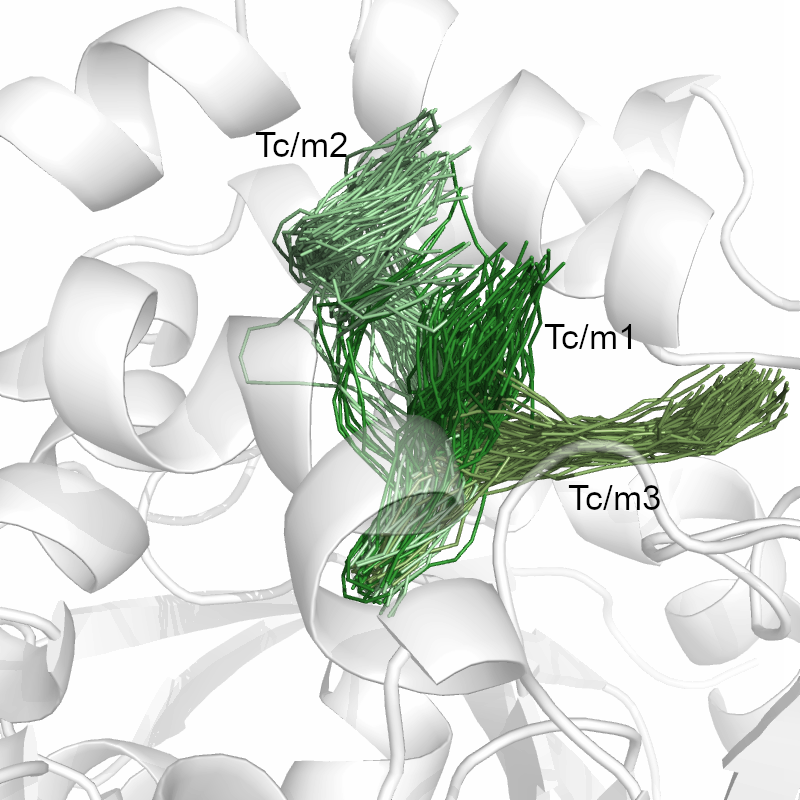

Supplement: S1 Fig — (TIFF) [file pcbi.1010119.s015.tiff]

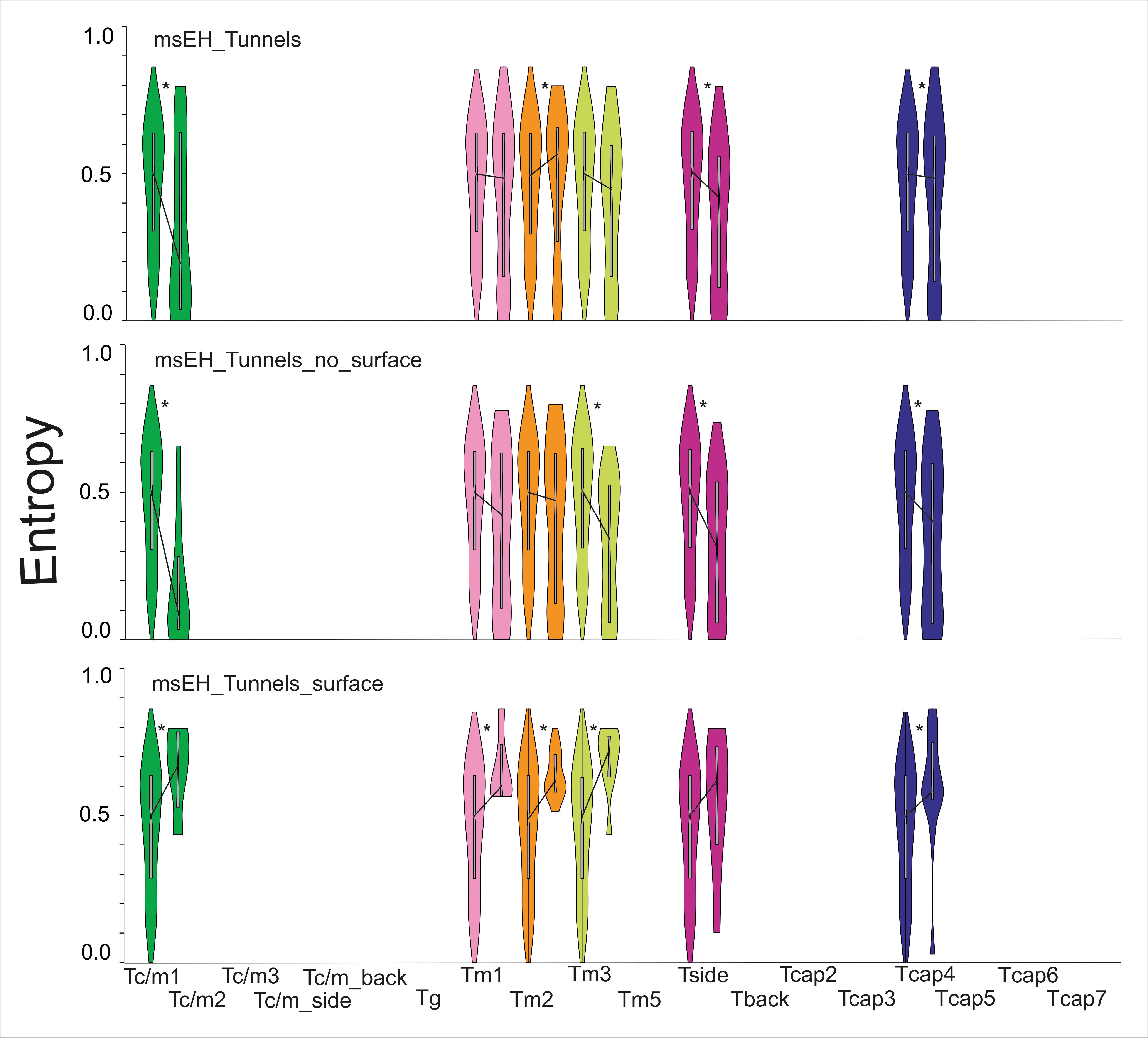

Supplement: S3 Fig — Statistically significant pairwise differences in the median distance values are marked by a star (*). (TIFF) [file pcbi.1010119.s017.tiff]

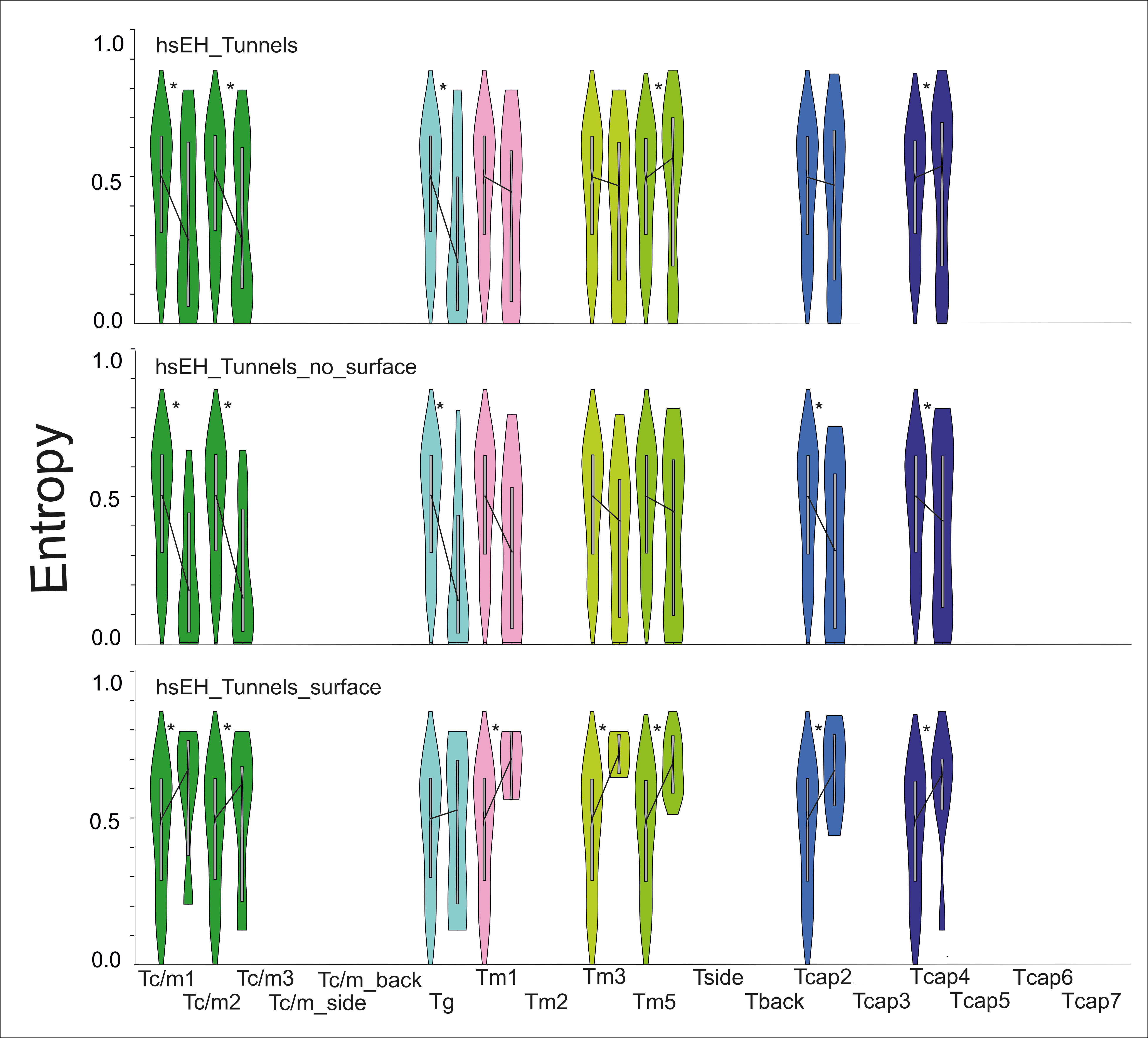

Supplement: S4 Fig — Statistically significant pairwise differences in the median distance values are marked by a star (*). (TIFF) [file pcbi.1010119.s018.tiff]

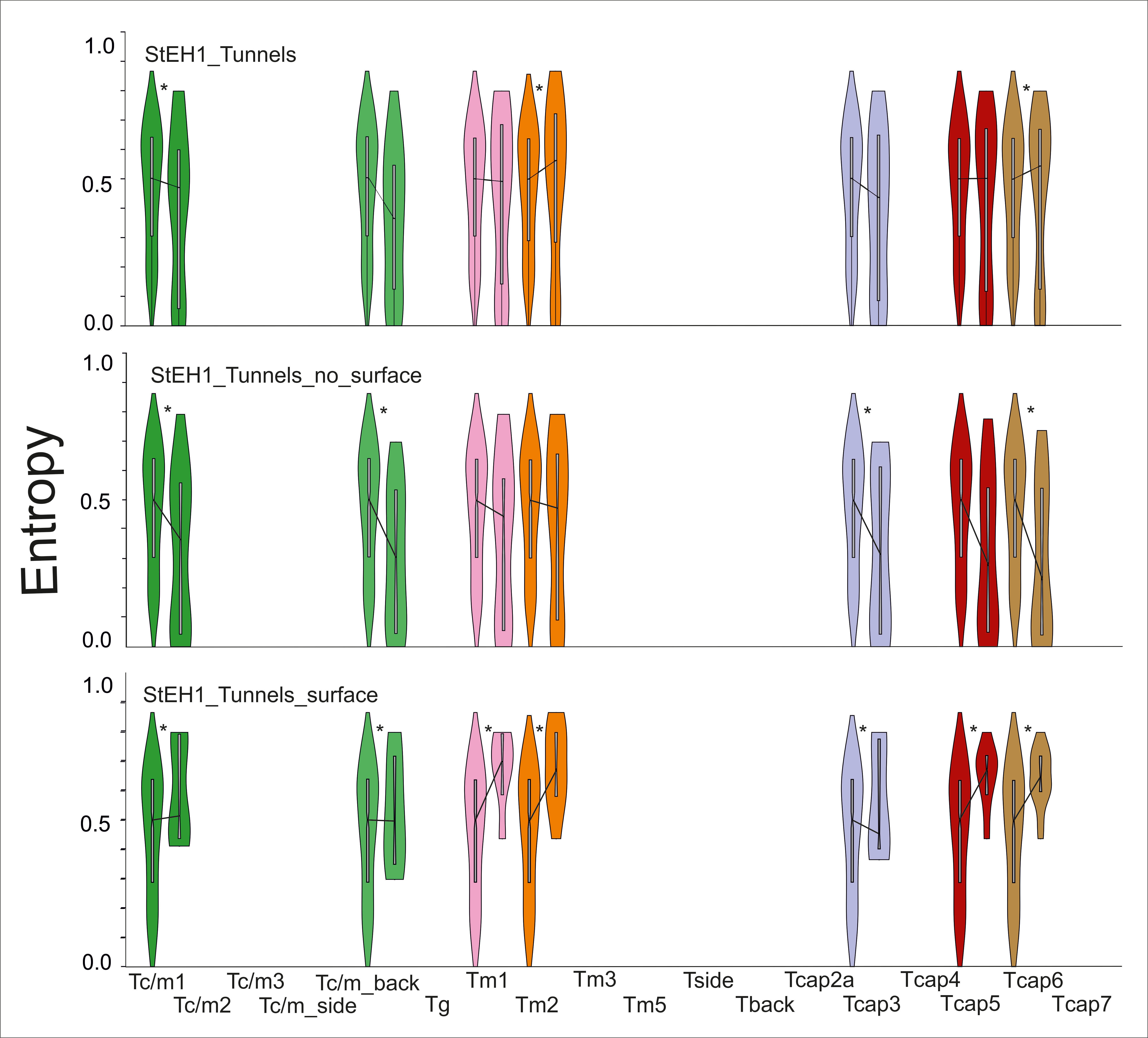

Supplement: S5 Fig — Statistically significant pairwise differences in the median distance values are marked by a star (*). (TIFF) [file pcbi.1010119.s019.tiff]

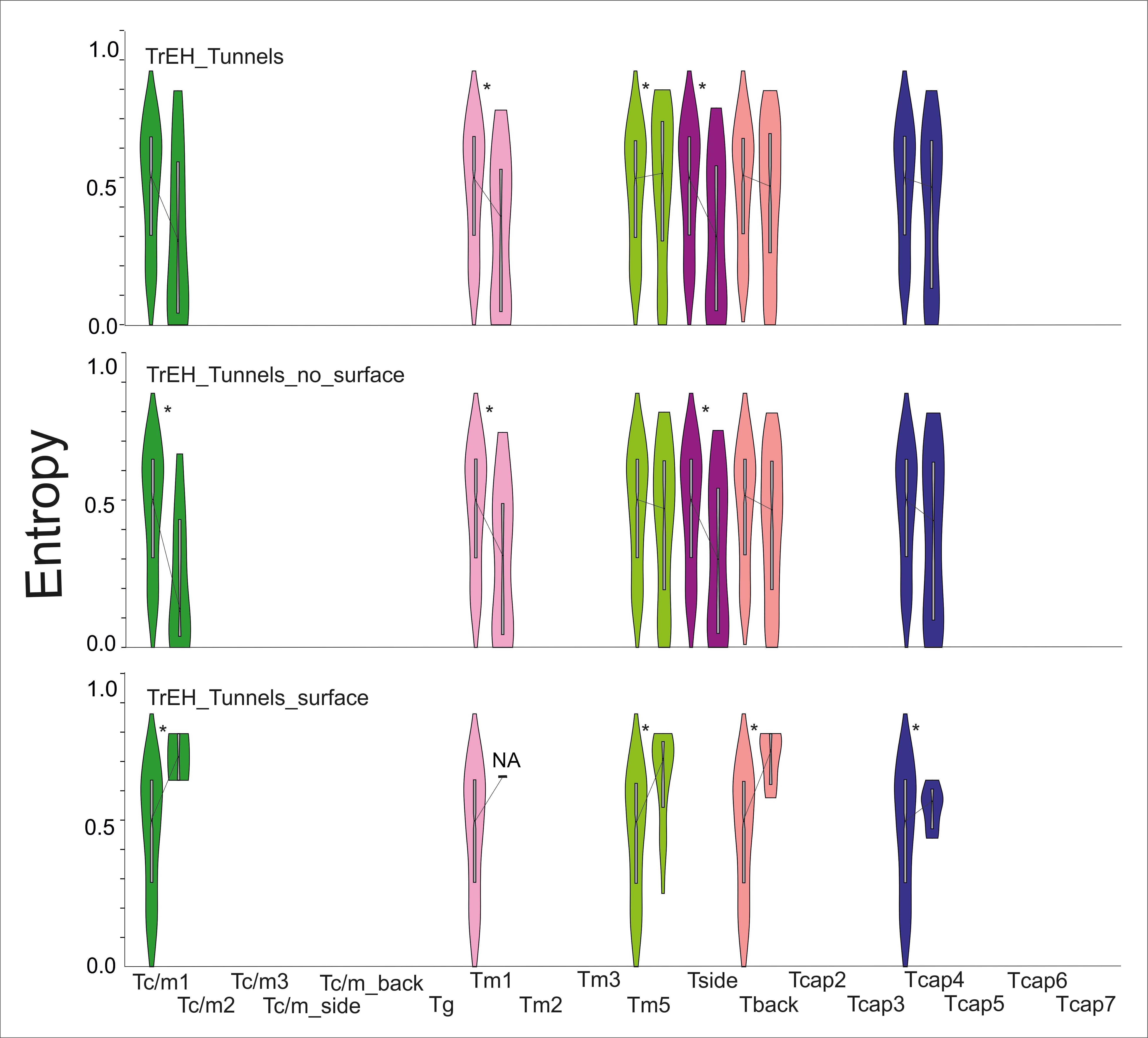

Supplement: S6 Fig — Statistically significant pairwise differences in the median distance values are marked by a star (*). NA by the violin plots means that the number of surface residues was insufficient to obtain the p-value of the Epps–Singleton two-sample test. In the case of the Tside tunnel, no surface residues were identified. (TIFF) [file pcbi.1010119.s020.tiff]

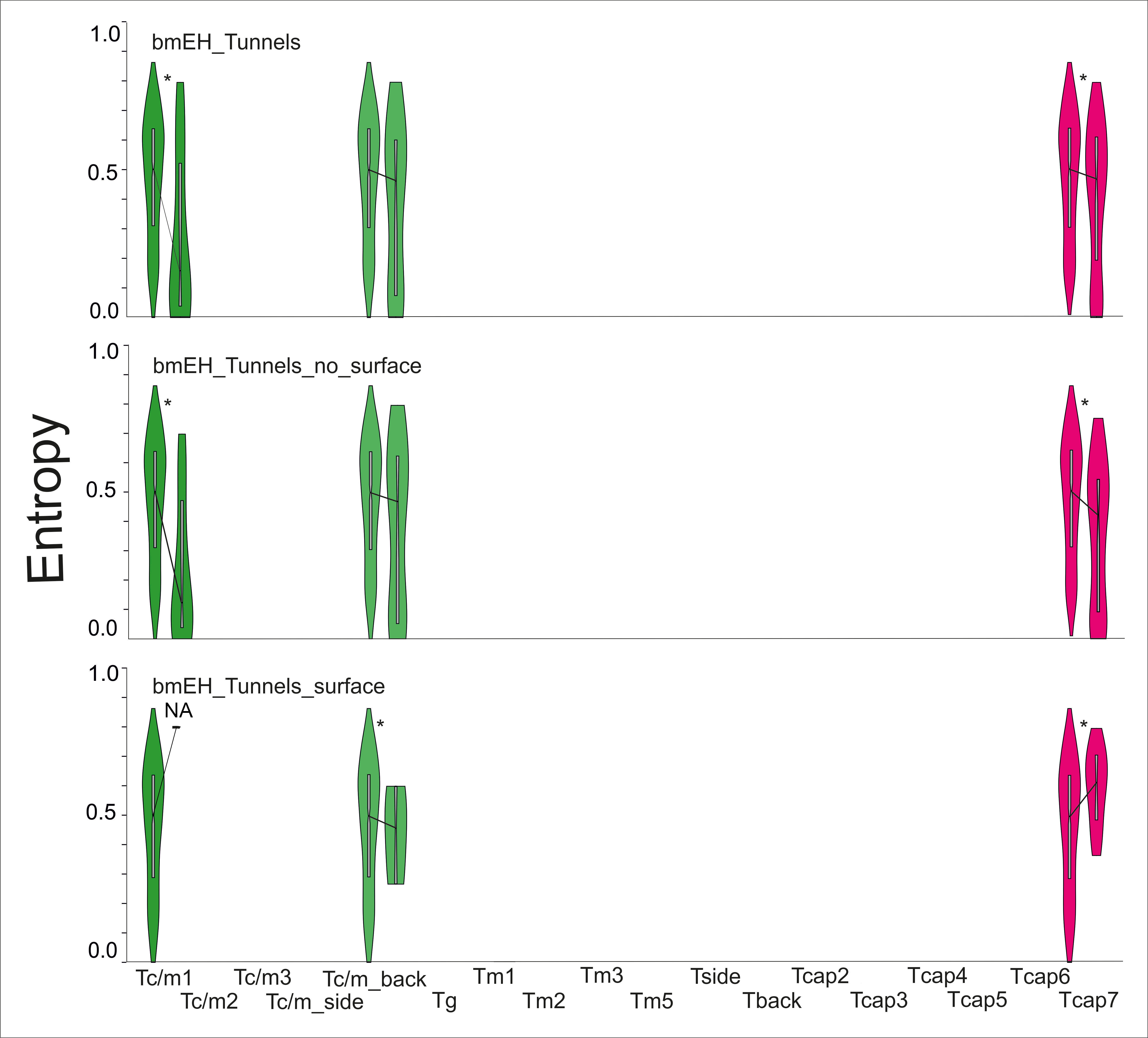

Supplement: S7 Fig — Statistically significant pairwise differences in the median distance values are marked by a star (*). NA by the violin plots means that the number of surface residues was insufficient to obtain the p-value median distance of the Epps–Singleton two-sample test. (TIFF) [file pcbi.1010119.s021.tiff]

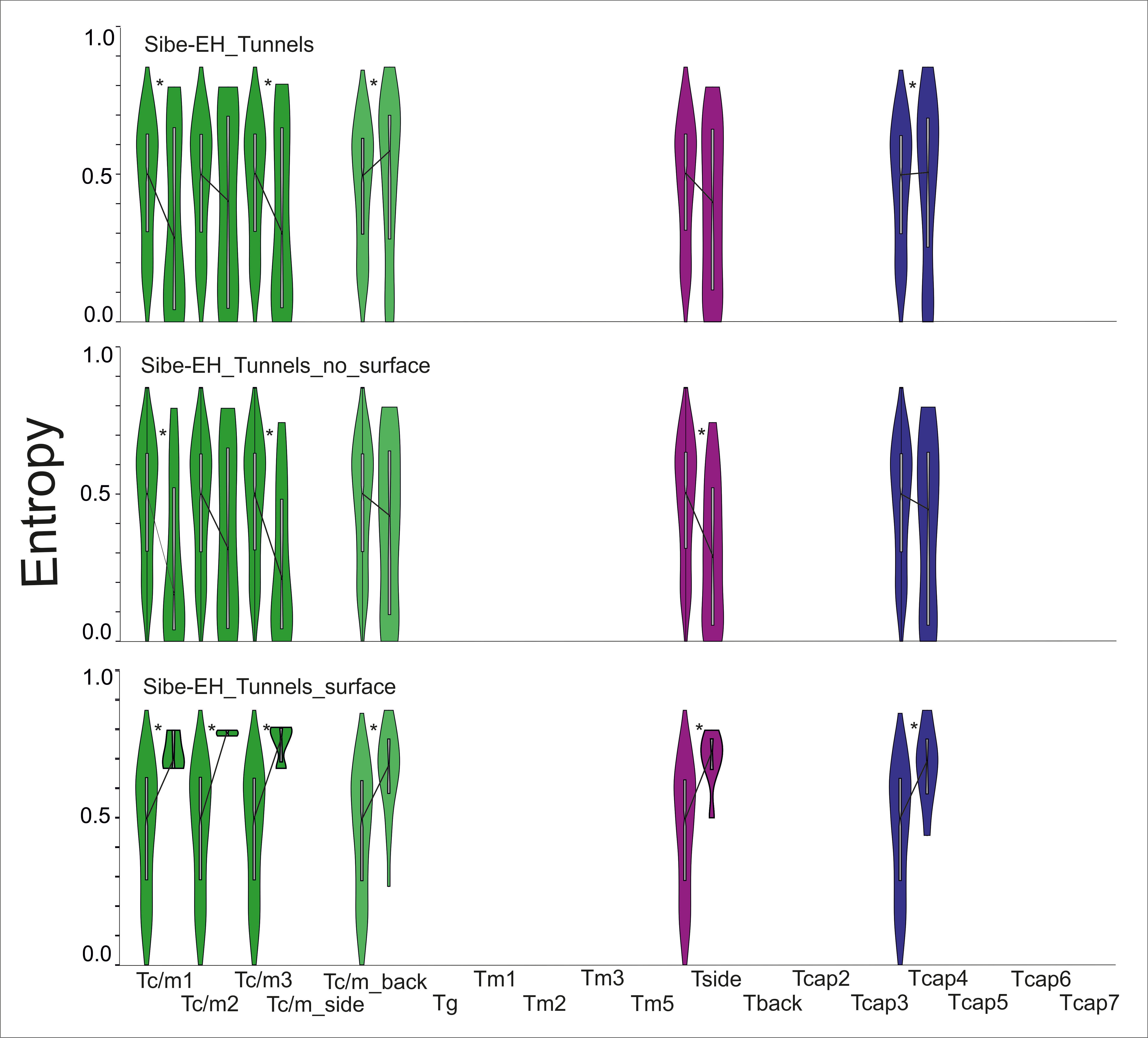

Supplement: S8 Fig — Statistically significant pairwise differences in the median distance values are marked by a star (*). (TIFF) [file pcbi.1010119.s022.tiff]

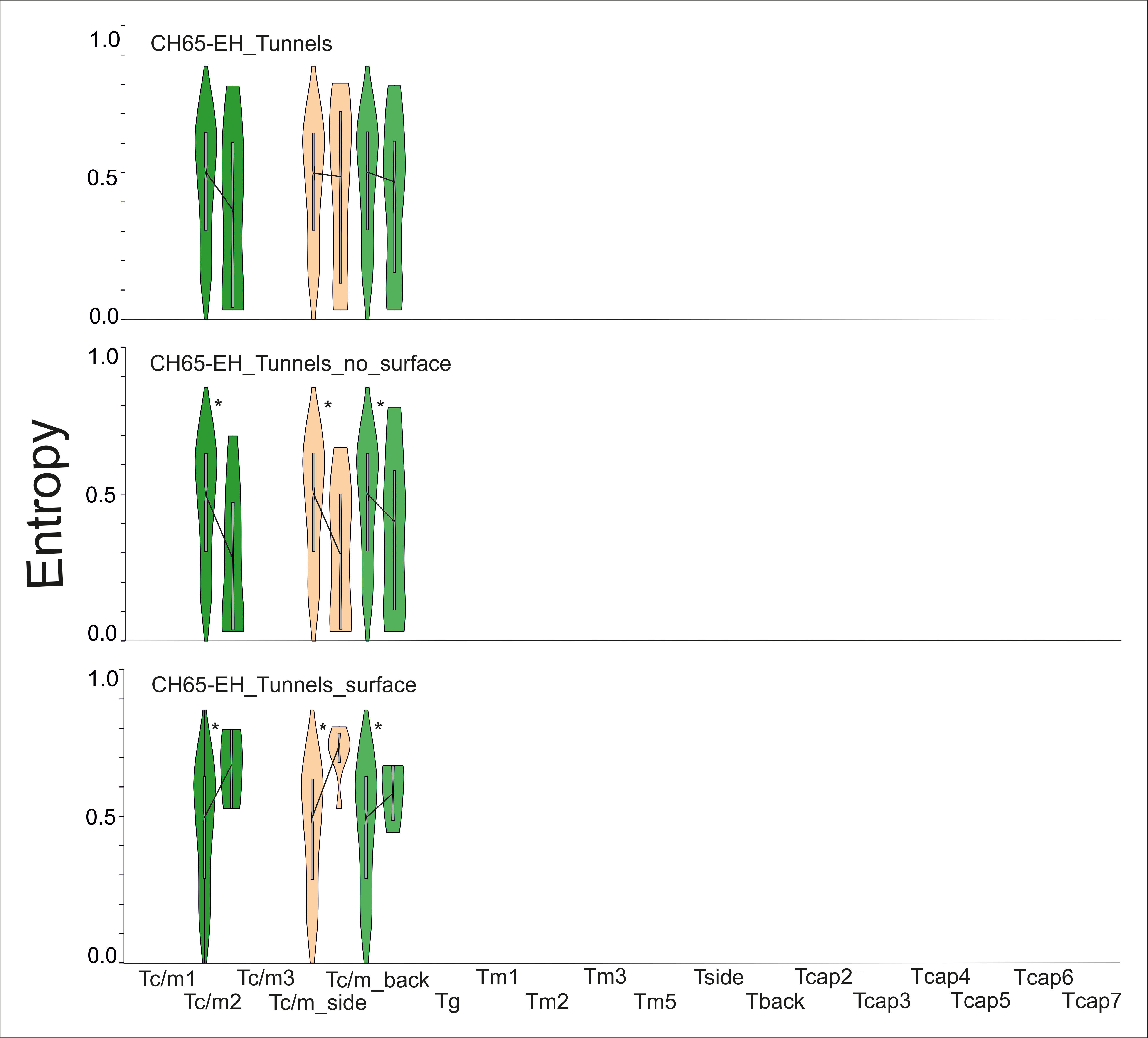

Supplement: S9 Fig — Statistically significant pairwise differences in the median distance values are marked by a star (*). (TIFF) [file pcbi.1010119.s023.tiff]

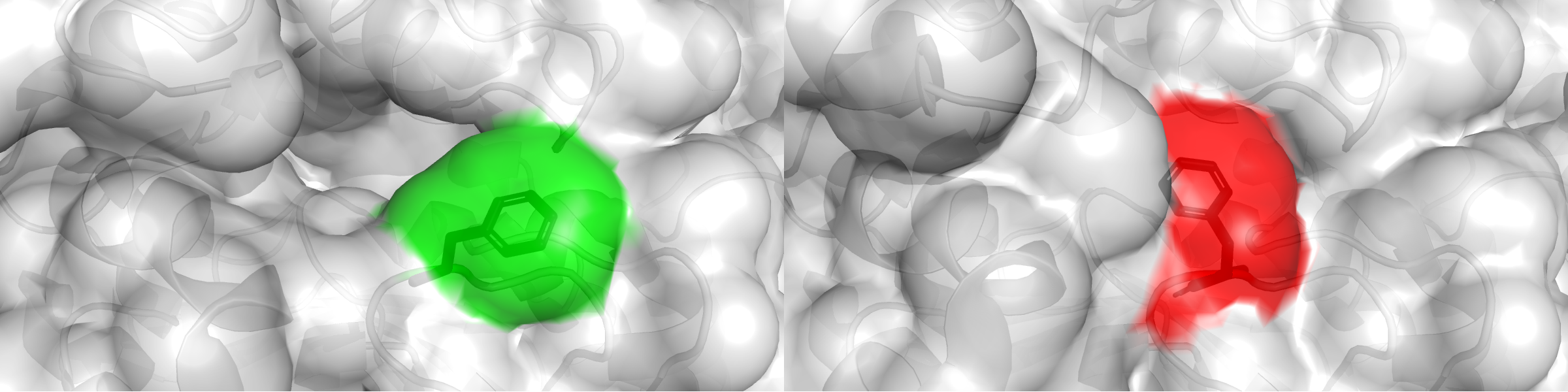

Supplement: S10 Fig — The protein is shown as cartoon and surface, and the F497 residue is shown as sticks. (TIFF) [file pcbi.1010119.s024.tiff]

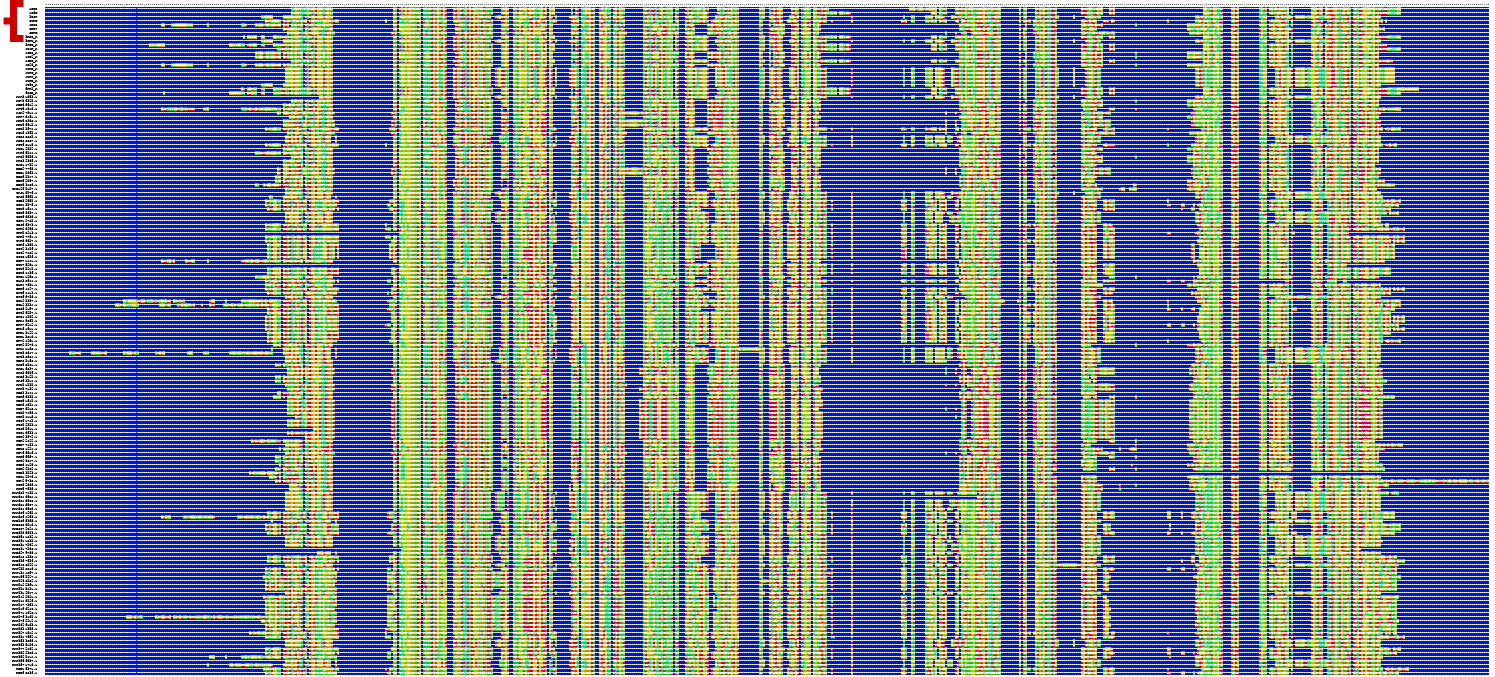

Supplement: S11 Fig — The red brace marks the sequences of the soluble epoxide hydrolases with known crystal structures. Gaps are marked in blue. MSA was pictured using the DECIPHER library for R (https://www.rdocumentation.org/packages/DECIPHER/versions/2.0.2). (TIFF) [file pcbi.1010119.s025.tiff]

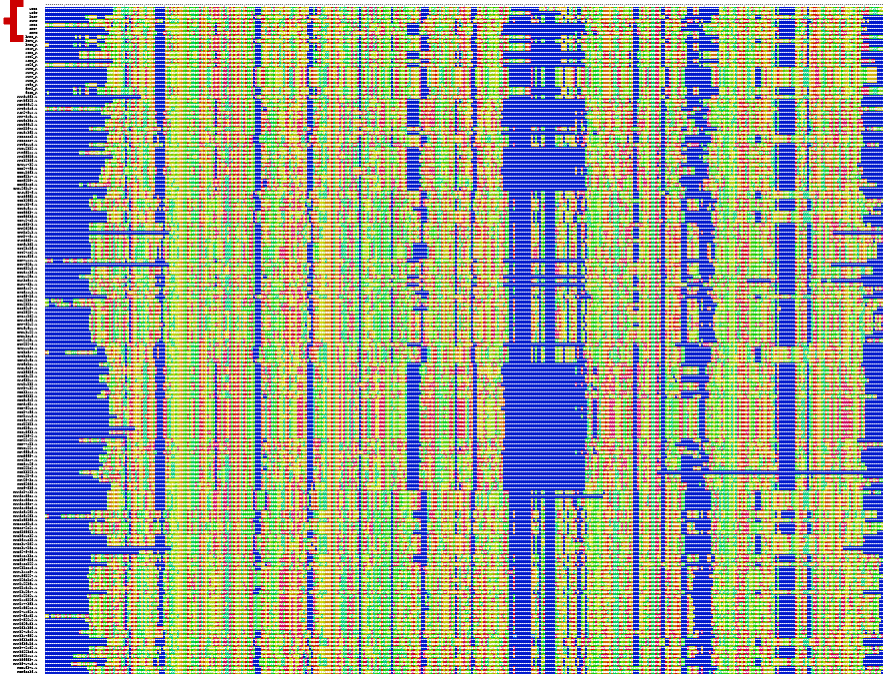

Supplement: S12 Fig — The red brace marks the sequences of the soluble epoxide hydrolases with known crystal structures. Gaps are marked in blue. MSA was pictured using the DECIPHER library for R (https://www.rdocumentation.org/packages/DECIPHER/versions/2.0.2). (TIFF) [file pcbi.1010119.s026.tiff]
